# Review on Damage and Failure in Adhesively Bonded Composite Joints: A Microscopic Aspect

**DOI:** 10.3390/polym17030377

**Published:** 2025-01-30

**Authors:** Sota Oshima, Jun Koyanagi

**Affiliations:** 1 Department of Aeronautics and Astronautics, Tokyo Metropolitan University, 6-6 Asahigaoka, Hino-shi 191-0065, Tokyo, Japan; 2 Department of Materials Science and Technology, Tokyo University of Science, 6-3-1 Niijuku, Katsushika-ku 125-8585, Tokyo, Japan; koyanagi@rs.tus.ac.jp

**Keywords:** adhesive joints, fracture mechanism, finite-element analysis, microscopy, composites

## Abstract

Adhesively bonded joints offer numerous advantages for industrial applications. However, because damage and failure in adhesively bonded joints occur within thin adhesive layers between stiff adherends, experimental characterization and numerical simulation that account for microscopic phenomena are particularly challenging. For adhesively bonded composite joints, in particular, the interaction between adhesive and adherend damage must also be carefully considered. This review article mainly discusses and reviews the microscopic aspects of damage and failure in adhesively bonded composite joints for aerospace applications. Three main topics are addressed in this article. First, the peculiar deformation and damage behaviors of polymeric materials, including their dependence on stress triaxiality, are discussed. Second, the experimental characterization of deformation and damage in adhesive layers using advanced microscale inspection techniques is reviewed. Lastly, the modeling and numerical simulation of damage and failure processes, incorporating microscopic phenomena, are explored. The article concludes with a discussion of future perspectives.

## 1. Introduction

**Figure 1:**
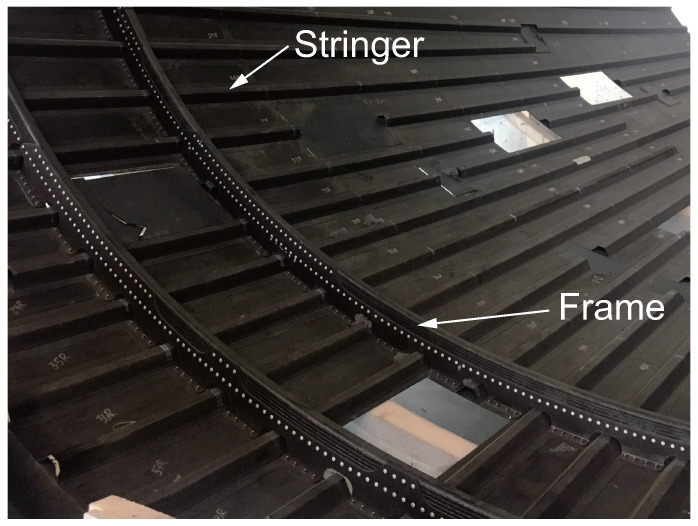
CFRP fuselage of Boeing 787.

Adhesively bonded joints offer many advantages, such as lightweight, avoidance of stress concentration around holes, and the ability to maintain the smooth surfaces of adherends, compared to mechanical joints [1,2]. Due to fiber discontinuity around holes and low bearing strength in composite mechanical joints, adhesively bonded joints are more suitable for composite structures than those made of metals. Despite these advantages, mechanical joints are still used alongside adhesively bonded joints due to concerns about their reliability. Figure 1 shows a carbon fiber-reinforced polymer (CFRP) fuselage of the Boeing 787. Stringers are attached to the skins solely with adhesives, while frames are connected to the skins with both bolts and rivets, in addition to adhesives. For aircraft structures, although arresting fasteners are often used to prevent excessive debonding in adhesively bonded joints, in accordance with the Advisory Circular of the Federal Aviation Administration, these fasteners may increase manufacturing costs [3,4,5,6,7].

Dimensional accuracy is also an important aspect of the reliability of adhesively bonded joints. The thickness of composite laminates is less precisely controllable during the manufacturing process compared to rolled metals. In addition, thermoset composites, which are commonly used for the primary structures of aircraft, cannot be shaped through post-processing, unlike metal plates. Although large monolithic components can be easily manufactured with composites, this also means that larger tolerances must be allowed in the joints. Because bondline thickness is a key factor in joint reliability [8,9], quality control in adhesively bonded joints in composite structures is significantly more challenging.

Since adhesive bonding is a long-established technique, various test methods have been proposed. In aerospace applications, shear and peel strengths are considered critical factors for adhesively bonded joints. Single lap joints (SLJs) and T-peel joints are traditionally used for shear and peel strength tests, respectively. Although these test methods are still used as standards, they cannot obtain pure shear and peel strengths due to factors such as stress concentration at edges, bending, and energy dissipation in adherends [10]. Therefore, comparisons under the same conditions may vary when using these methods.

Evaluation under fatigue loading is also crucial for adhesively bonded joints. For instance, aircraft fuselages undergo one fatigue cycle per flight due to pressurization. Certain industrial applications, such as wind turbines, are designed to endure high-cycle fatigue (exceeding 100 million cycles) [11]. Similar to metals, the fatigue strength of polymers generally decreases with an increasing number of cycles. However, the fatigue strength of polymers is more sensitive than that of metals due to self-heating effects. During cyclic tests of polymers, hysteresis loops in stress–strain relations result in energy dissipation, which induces self-heating effects [12]. Because of the relatively low thermal conductivity of polymers, self-heating effects are more pronounced in adhesively bonded composite joints [13]. The fatigue limit is influenced by the temperature rise caused by self-heating, which can lead to differences in the coefficients of thermal expansion (CTEs) between adherends and adhesives, polymer degradation, and material softening. Additionally, since heat generation depends on the strain rate of polymers, the test frequency significantly affects the fatigue life of adhesively bonded joints.

Fracture mechanics enables the quantitative evaluation and modeling of failure in adhesively bonded joints. Griffith formulated the energy balance associated with crack growth [14]. In the energy-based approach of fracture mechanics, crack propagation occurs when the energy overcomes the material resistance [15]. Thus, this approach allows for the quantitative evaluation of crack propagation. It has been firmly supported by engineers because crack propagation behavior can be effectively modeled in numerical simulations. Damage and fracture behaviors are often modeled using the virtual crack closure technique, cohesive zone modeling (CZM), or the J-integral. Among these methods, the CZM has been widely adopted for progressive damage analysis of adhesively bonded joints because it can model plastic and damage behaviors with a relatively simple formulation while avoiding stress singularity at the crack tip. In the CZM, the energy dissipated until final failure equals the fracture toughness, provided no energy dissipation occurs in the adherends. However, since the CZM relies only on the resultant behavior during crack growth, its properties must be evaluated based on specific joint conditions (e.g., bondline thickness, type of adherends, mixed-mode ratio, and other bonding parameters).

Another approach to damage modeling of adhesively bonded joints is the continuum mechanics approach. Although the fracture mechanics approach assumes the presence of a singular stress field, the damage mechanics approach is more applicable to tough and ductile adhesives because it does not rely on a stress singularity field. Stress triaxiality (i.e., the ratio of volumetric stress to equivalent stress) is often considered in the damage mechanics approach for structural adhesives. In contrast to the CZM, variations in adhesive thickness and mixed-mode ratio effects can be simulated using consistent material properties. However, determining complex material properties remains a significant challenge for the damage mechanics approach.

The bondline thickness depends on the specific applications of adhesively bonded joints. In the aerospace industry, the bondline thickness is typically set between 0.1 and 0.5 mm, considering the trade-off between shear and peel strengths [16]. In contrast, due to the relatively lower manufacturing tolerances of large components in the wind energy industry, thicker bondlines in the range of 5 to 30 mm are employed, typically using filled epoxy or methyl methacrylate adhesives [11,17,18,19,20,21]. Although the failure behavior of adhesively bonded joints depends on the bondline thickness, this review article primarily focuses on relatively thin bondlines, such as those using epoxy adhesives for aerospace structures, where microscopic aspects are more critical. Furthermore, thin bondlines are more constrained by the adherends compared to thicker adhesives. For thin bondlines, in particular, characterizing damage and failure behaviors is challenging because a thin yet long damage zone tends to form within the adhesive layers. This unique damage zone formation also makes the microscopic characterization of adhesively bonded joints more complex.

Stress triaxiality is a key parameter influencing damage in adhesively bonded joints. The yield and damage behaviors of polymeric materials are highly sensitive to stress triaxiality. Stress triaxiality-dependent behavior contributes to the size effect on the strength of polymeric materials. Under high-stress triaxiality, polymeric materials exhibit lower yield stress and brittle fracture, whereas they become ductile under low-stress triaxiality (e.g., under shear or compressive loading) [22,23,24]. Surprisingly higher ultimate stress and strain at failure are observed in microfiber of resin or at the bottom of a notch under tensile loading due to the size effect [25,26,27]. Therefore, stress triaxiality-dependent behavior must be taken into account to accurately understand and model damage and fracture behaviors in adhesively bonded joints.

This review article focuses on three main topics. The first section discusses the deformation and damage behaviors of polymeric materials used in adhesively bonded joints. The second section presents the experimental characterization of damage accumulation in adhesively bonded composite joints at the microscale. Finally, the third section reviews the modeling and numerical simulation of damage behaviors in adhesively bonded composite joints.

## 2. Stress Triaxiality-Dependent Behavior of Polymeric Materials

**Figure 2:**
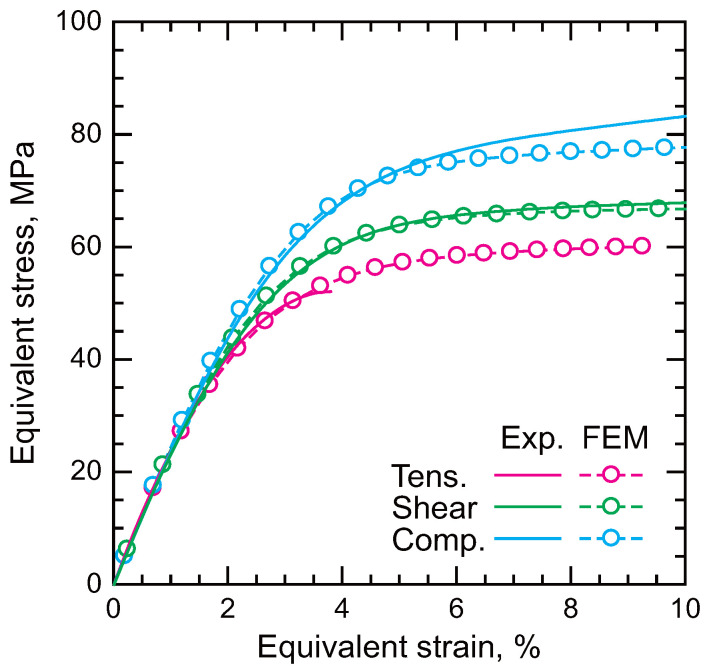
Stress–strain curves of epoxy adhesive with different types of loading [28].

The mechanics of materials has been primarily developed based on the deformation and damage behaviors of metals. However, the deformation and damage behaviors of polymers differ significantly from those of metals. For example, the von Mises criterion is a well-known and fundamental yield criterion. In this criterion, only the deviatoric stress components influence yielding. While this model accurately describes yielding in metals, hydrostatic stress also affects the yielding of polymers. Figure 2 illustrates the equivalent stress–equivalent strain curves of an epoxy resin under different types of loading [28]. Stress triaxiality increases as the types of loading change from compression to shear and then to tension. The yield stress decreases with increasing stress triaxiality [22,23,24,28,29,30]. Plots from finite element method (FEM) results are explained in a later section on modeling (Section 4). Due to the constraint imposed by adherends, stress triaxiality in adhesive layers increases further compared to bulk polymers under out-of-plane tension [23]. Consequently, failure under out-of-plane tension or mode I (opening) loading often occurs in a brittle manner in adhesively bonded joints.

Strain at failure is also influenced by stress triaxiality, as shown in Figure 2. For bulk epoxy resins, the strain at failure under tensile loading is typically within a few percent, whereas under shear loading, it exceeds 10% [24]. Fracture under tensile loading is usually initiated from defects, whereas more ductile tearing behavior is often observed under shear loading. This phenomenon indicates a ductile-to-brittle transition in polymers. A similar ductile-to-brittle transition is also observed in adhesively bonded joints, depending on the types of loading.

Since the tensile failure of epoxy resins occurs in a brittle manner, the strain at failure depends on the volume of the resin. The strain at failure increases as the volume decreases [24]. Although epoxy resins are generally considered a brittle material, failure strains ranging from 20% to 45% under tension have been reported for epoxy microfibers [26]. Strength also increases with decreasing volume [25,26]. Failure stresses 1.3 to 2 times higher than those of bulk epoxy resins have been observed for epoxy microfibers [25,26]. Despite the surprisingly ductile behavior exhibited by epoxy microfibers compared to bulk epoxy resins, failure in microfibers is still initiated by defects in the resin [25]. Since crack propagation is a localized phenomenon occurring near crack tips, the volume effect is also found in fracture toughness specimens. For example, a comparison of single-edge notched bending specimens made of a neat epoxy resin with sharp and blunt cracks revealed that the strength of epoxy resins at crack tips (i.e., in localized areas) can be six to ten times higher than that measured in bulk polymers [27].

Because thin adhesive layers are constrained by stiff adherends, the stress states in the adhesive layers differ from those of bulk polymers. For instance, adhesive layers under out-of-plane tension tend to shrink in the in-plane direction due to the Poisson effect. However, this shrinkage is restricted by the presence of the adherends. As a result, higher stress triaxiality is typically observed in the adhesive layers due to the triaxial stress state [29]. In two-dimensional problems, the stress state of adhesively bonded joints can often be approximated as plane strain. Differences in CTEs between adherends and adhesives further increase stress triaxiality in heat-cured adhesives because the CTEs of polymers are generally higher than those of adherend materials such as composites and metals. Additionally, chemical shrinkage occurs during the curing process, contributing to the stress state. Stress distribution under varying mixed-mode ratios indicates that the highest stress triaxiality is observed under pure mode I loading in fracture toughness specimens, while it significantly decreases with an increase in the mode II (shear) component [29].

The composition of polymers is, of course, a crucial factor influencing their mechanical properties. For instance, urethane and acrylic adhesives are typically more flexible than epoxy adhesives. Different stress–strain curves have been reported depending on the specific type of epoxy resins used [26]. Because epoxy resins are relatively brittle, various toughening strategies, such as polymer alloying and rubber modification, have been implemented [31,32,33,34]. The toughening mechanism of rubber-modified adhesives is closely related to stress triaxiality. When high hydrostatic stress is applied to rubber-modified epoxy resin, a significant number of free surfaces, so-called cavities, are formed, as reported in previous studies [29,35,36,37]. Cavities change the stress state from one where plane strain is dominant to one where plane stress is dominant (i.e., reduction in stress triaxiality), thereby inducing shear yielding in the epoxy resin [35]. Once cavities occur, the epoxy resin is toughened through energy dissipation during plastic deformation. Although numerous factors influence the damage and failure behaviors of polymers, this review article primarily focuses on the mechanical aspects.

## 3. Experimental Characterization

Microscopic characterization of deformation and damage behaviors is conventionally performed using ex situ methods such as scanning electron microscopy (SEM), transmission electron microscopy (TEM), and atomic force microscopy (AFM). Thanks to extensive studies employing these methods, various damage mechanisms have been elucidated. For instance, toughening mechanisms in rubber-modified adhesives have been discussed based on observations made with these microscopy techniques. Numerous cavities are visible on the fracture surface of mode I fracture toughness specimens bonded with rubber-modified epoxies, as observed by SEM and AFM [29,38,39,40]. The distribution of cavities has also been characterized using TEM images [39,40,41,42]. These techniques are effective for capturing the deformation and damage behaviors in adhesively bonded joints. However, microscopy observation methods generally only evaluate the resultant failure morphologies because preprocessing is required prior to observation. Thus, such observation techniques are primarily performed after unloading [43], and fewer in situ observations have been reported. Although some researchers have utilized in situ SEM systems to characterize damage processes, the specimen shape is highly constrained due to the placement of the microtester inside the SEM chamber [44,45].

With the development of in situ techniques, not only the resultant failure morphology but also the damage progression has been characterized. In situ techniques can be categorized into direct damage observation methods (e.g., optical microscopy, SEM, and thermoelastic stress analysis) and indirect damage detection methods (e.g., acoustic emission (AE) testing). Indirect detection methods monitor damage progression using relatively simpler systems. AE testing evaluates stress waves associated with damage accumulation. Since the peak frequency, duration, amplitude, and other parameters of stress waves depend on the types of damage, AE testing has been widely adopted for real-time damage monitoring [28,46,47,48]. However, correlating specific types of damage with indirect detection methods is challenging because damage progression is not directly observed. Additionally, determining the exact damage location at the microscale is difficult with indirect methods, although rough estimations of damage location can be achieved using multiple AE sensors.

Crack length during fracture toughness tests provides valuable information about damage progression. Since crack length is necessary to calculate the strain energy release rate, several methods for measuring crack length have been proposed to date. Although crack length can be directly measured through in situ optical observation, some indirect methods have been developed because continuous measurement of crack length is challenging. Compliance-based crack length measurement is widely used to determine the effective crack length and has been incorporated into standard test methods [49,50,51,52]. The effective crack length obtained from compliance reflects not only the actual crack propagation but also changes in compliance caused by damage progression, large deformations, shear stresses, and friction [53,54]. As a result, R-curves derived from the effective crack length provide insight into the extent of damage progression, particularly before actual crack propagation occurs. Additionally, because adhesive layers are more flexible than adherends, the effects of local deformation around the crack tip have been also considered in analysis [55]. Image processing has also been increasingly used for crack length measurement. Because deflection along the longitudinal direction of the beams can be obtained through image processing, the crack tip location can be determined more precisely using image-based measurement, although high-resolution digital images are required [56,57].

Although indirect methods can continuously evaluate damage progression, characterizing damage within the fracture process zone remains challenging. To correlate damage behavior with macroscopic strength criteria (e.g., strain energy release rate), direct microscopic observations of fracture toughness specimens have been performed. Recent advancements in optical measurement techniques have enabled more detailed characterization of microscopic failure mechanisms. Hereafter, we focus on the microscale characterization of damage progression within the fracture process zone, particularly considering the effects of stress triaxiality.

**Figure 3:**
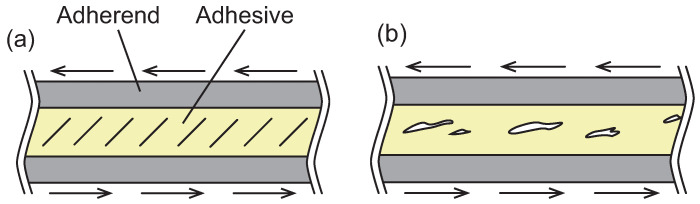
Fracture behavior of adhesive depending on ductility under mode II loading. (**a**) Brittle adhesive. (**b**) Ductile adhesive.

**Figure 4:**
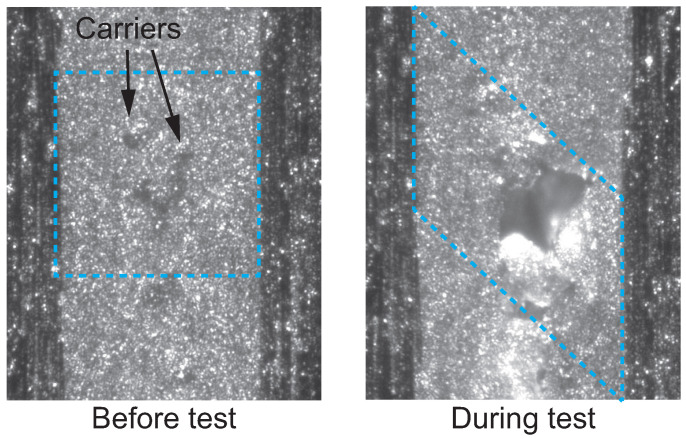
Micrograph of ductile tearing under mode II loading [62]. The dashed frames show the average shear deformation within the adhesive layer.

Under mode II loading, in situ optical observation of damage processes around the crack front in adhesively bonded joints have been carried out using optical microcopy [54,58,59,60,61,62]. The results of microscopic observation revealed that microcracks initially appear ahead of the crack tip and then coalesce. The shape of these microcracks depends on the ductility of the adhesive, as illustrated in Figure 3. For brittle adhesives, microcracks propagate in a direction perpendicular to the principal normal stress [54,58,59]. Similar microcracks are also observed in the interlaminar regions of polymer matrix composite laminates [53,63]. In contrast, ductile tearing occurs as the ductility of adhesives increases [60,61,62]. Figure 4 shows a micrograph of ductile tearing [62]. The ductile tearing initiates from a nonwoven fabric carrier embedded in the film adhesive. It is noteworthy that significant deformation occurs within the adhesive layer, as indicated in Figure 4. The carrier is introduced to maintain the shape of the adhesive film during stacking and to ensure a minimum bondline thickness. Although the carrier may contribute to the initiation of ductile tearing, it also prevents interfacial crack propagation between the adherends and the adhesive. By intentionally controlling the initiation sites of microcracks, the carrier can mitigate sudden drops in fracture toughness during crack propagation. Theoretical discussions on changes in microcrack shape based on the yield stress of adhesives have also been presented [64].

Recent advancements in measurement techniques have enabled the quantitative characterization of deformation and damage processes. Quantitative data are also valuable for comparison with numerical simulation results and for model construction. The digital image correlation (DIC) [29,62,65,66,67,68,69,70] and sampling moiré methods [71,72,73] are widely used to quantify deformation and damage processes. Among these methods, The DIC method has gained increasing popularity because it is simpler than the sampling moiré method for obtaining two-dimensional deformation and strain fields from digital images.

**Figure 5:**
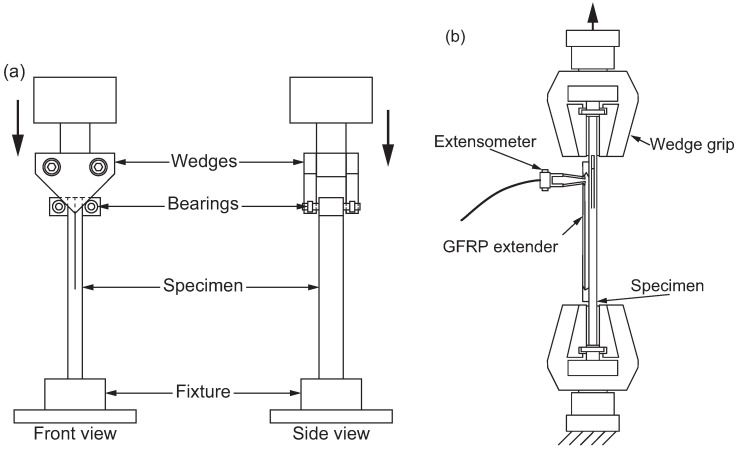
Fracture toughness tests suitable for stationary observation. (**a**) Wedge-loaded double cantilever beam (DCB) specimen [74]. (**b**) Doubly end-notched tension (DENT) specimen [76].

Stationary observation is essential for applying the DIC method to digital images. However, achieving stationary observation in adhesive layers at the microscale is challenging because rotational movements often occur when using standard test methods such as double cantilever beam (DCB) and end-notched flexure (ENF) specimens. To enable high-magnification observation, several alternative test methods have been proposed. Figure 5 illustrates the wedge-loaded DCB specimen for mode I loading [74,75] and the doubly end-notched tension (DENT) specimen for mode II loading [76,77], both of which are suitable for high-magnification observation without rotational movement.

The DIC method determines deformation by analyzing the contrast on specimen surfaces. To create this contrast, random speckle patterns are typically applied by spraying paint. However, the speckle patterns generated by spraying can obscure the failure morphology during testing. One solution is to enhance surface roughness visibility using oblique illumination, as shown in Figure 4. When specimens are properly polished, oblique illumination can provide sufficient contrast for the DIC method without the need for sprayed speckle patterns.

**Figure 6:**
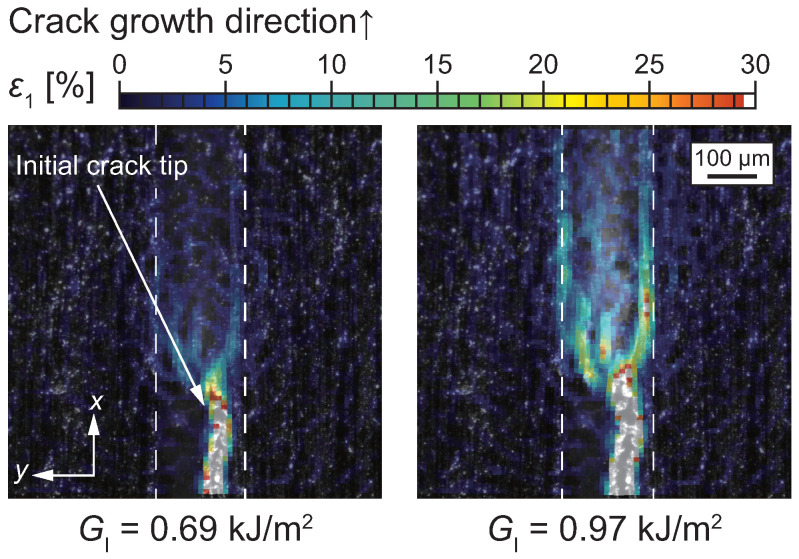
Maximum principal strain distribution under mode I loading analyzed by DIC method [62].

**Figure 7:**
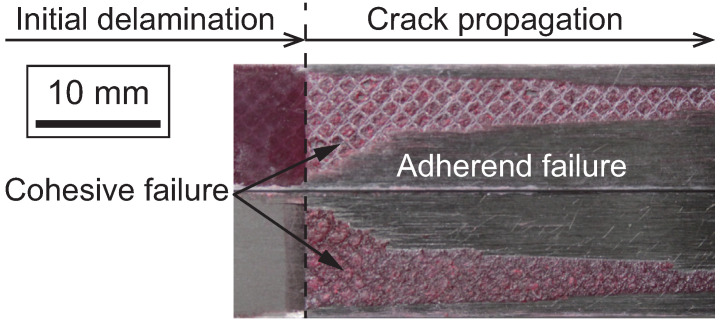
Fracture surfaces of adhesively bonded CFRP joints under mode I loading (adopted from [78]).

Figure 6 illustrates microscopic strain fields in the vicinity of the crack tip under mode I loading [29]. Under mode I loading, strain concentration was observed at the interface between the adhesive and the adherends. This strain concentration occurs because the adherends constrain the shrinkage of the adhesive layer in the width direction. As a result, the crack propagated along the interface region, often causing adherend (composite) failure. In contrast, X-ray computed tomography (X-CT) analysis revealed that, away from the specimen edges, the crack primarily propagated within the adhesive layer (i.e., cohesive failure), indicating no significant strain concentration along the interface [29]. However, as crack propagation continues, the ratio of adherend failure could increase, as shown in Figure 7, due to the relatively lower fracture toughness of the composite compared to the adhesive resin [78]. The interaction between adhesive and composite damage plays a critical role in the failure mechanisms of adhesively bonded composite joints.

**Figure 8:**
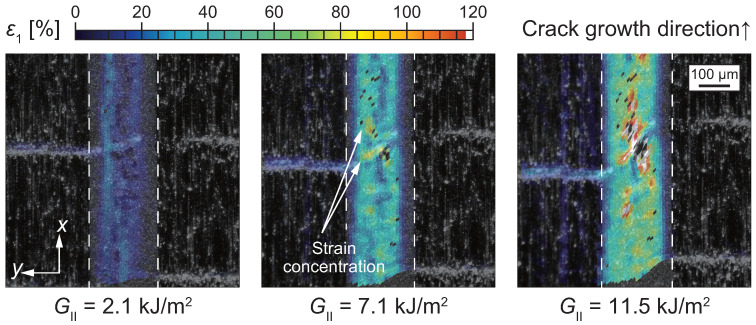
Maximum principal strain distribution under mode II loading analyzed by DIC method [62].

Figure 8 illustrates microscopic strain fields located 0.9 to 1.6 mm away from the initial crack tip under mode II loading [29]. As previously described in Figure 4, ductile tearing was initiated from the nonwoven fabric carrier embedded in the adhesive layer. The strain distribution further indicates that strain concentration occurs around the carrier due to the modulus difference between the carrier material and the epoxy resin. Under mode II loading, the equivalent strain exceeded 90% before final failure, in contrast to less than 30% observed under mode I loading. This significant difference arises from the relatively lower stress triaxiality under mode II loading, which enhances the deformability of the adhesive.

**Figure 9:**
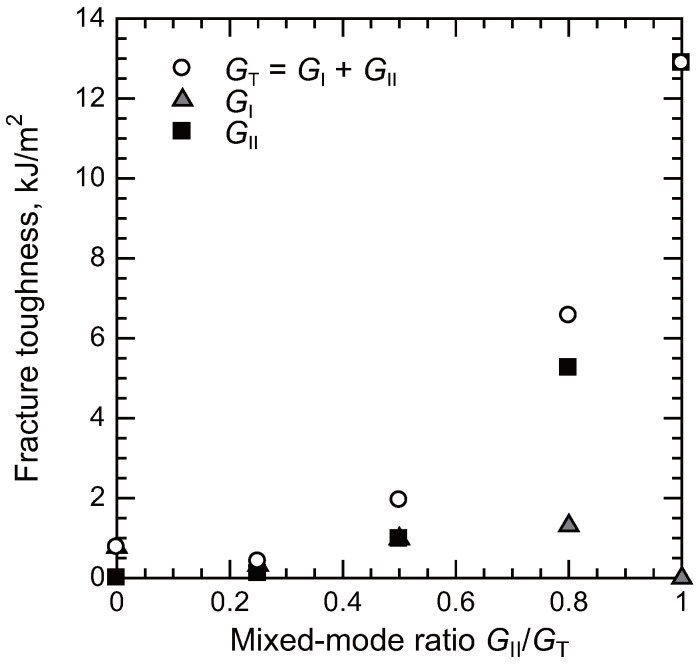
Fracture toughness of adhesively bonded composite joints depending on mixed-mode ratios [29].

The fracture toughness as a function of the mixed-mode ratio ($G_{\mathrm{II}}/(G_{I}+G_{\mathrm{II}}$)) is summarized in Figure 9 [29]. For the material system used in the reference study (NB102, Mitsubishi Chemical Carbon Fiber and Composites), the lowest fracture toughness was observed at a mixed-mode ratio of 0.25. Under pure mode I loading, the fracture toughness showed a slight increase. As the mode II component increased, the fracture toughness exhibited a significant rise, with values under mode II loading exceeding those under mode I loading by more than a factor of 10. This trend is consistent with observations from another toughened epoxy system [79]. The difference can be attributed to distinct toughening mechanisms operating under mode I and mode II loading conditions.

As described in Section 2, epoxy adhesives are often toughened by the addition of rubber particles. Under high-stress triaxiality, numerous free surfaces, referred to as cavities, are formed around the rubber particles [29,35,36,37,80]. These cavities transition the stress state from plane strain dominance to plane stress dominance, facilitating shear yielding in the modified epoxy resin [35,80]. Consequently, the epoxy resin is toughened by the energy dissipation that occurs during plastic deformation under a plane stress-dominated state. Since cavity formation is triggered by a certain level of hydrostatic stress [81,82], the toughening mechanism is also influenced by the mixed-mode ratio. In particular, under mode I loading, the toughening mechanism can be explained by the formation of cavities.

**Figure 10:**
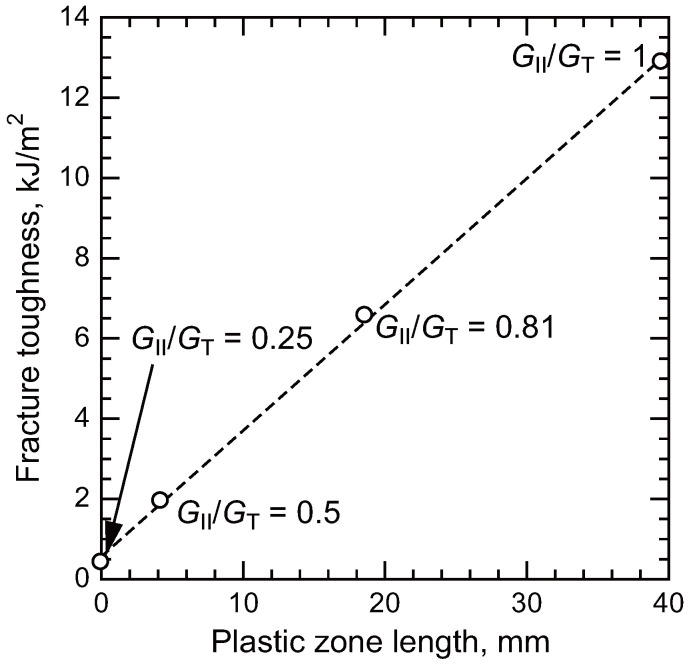
Relationship between fracture toughness and plastic zone length [29].

The increase in fracture toughness under mode II loading is primarily attributed to plastic energy dissipation within the adhesive layer. Since the failure behavior of polymeric materials depends on stress triaxiality, as discussed in Section 2 [22,23,24,28], an increase in mode II components during fracture toughness tests results in a decrease in stress triaxiality. This reduction in stress triaxiality leads to an increase in the failure strain under mode II loading. Figure 10 illustrates the relationship between fracture toughness and residual shear strain measured by the DIC method. A nearly linear correlation is observed in Figure 10, indicating that plastic deformation is the primary factor contributing to the enhanced fracture toughness under mode II loading.

Although optical observation of microscopic damage using image processing provides valuable insights into the microscopic damage process, other advanced techniques have also been employed. Mechanoluminescent (ML) materials are functional ceramic powders that emit light when subjected to applied stress. By dispersing ML powders into adhesive resins, the fracture process zone can be visualized and analyzed more effectively. ML materials have been increasingly used to identify areas of stress concentration, fracture process zones, and crack tip locations under both static and cyclic loading [83,84,85,86].

Thermoelastic stress analysis (TSA) has also been utilized to detect stress concentration areas in adhesively bonded joints [87,88]. As mentioned in the Introduction (Section 1), self-heating in adhesive resin typically has a negative effect on the mechanical properties of adhesively bonded joints. However, the self-heating effect can be beneficial for TSA. Previous studies have demonstrated that damage, such as debonding, can be effectively identified using TSA [87,88].

Although optical microscopy observes damage behavior on the surfaces of joints, X-CT can evaluate damage behavior within the materials. Synchrotron radiation (SR) X-CT is particularly suitable for microscopic damage evaluation due to its ability to achieve submicron resolution. To evaluate damage progression inside materials, in situ SR X-CT systems have been employed [89,90,91]. The digital volume correlation (DVC) method, which combines the DIC method with X-CT, enables the quantitative evaluation of deformation and damage behaviors within materials. Several researchers have reported quantitative damage evaluations of composite materials using this technique [92,93]. Although studies focusing on adhesively bonded joints are still limited, further insights into damage behavior within such joints are anticipated.

## 4. Modeling and Numerical Simulation

As experimental characterization reveals, stress triaxiality-dependent yield and damage behaviors are critical aspects of failure in adhesively bonded joints. Various yield functions that incorporate stress triaxiality have been proposed and utilized, including the Drucker–Prager [28,29,94,95,96,97,98,99,100,101,102], Mohr–Coulomb [103,104,105,106], and Gurson–Tvergaard–Needleman models [107,108,109,110,111,112]. For example, the yield function *F* of the linear Drucker–Prager yield criterion is given by [113].

(1) F=q−ptanβ−d=0,

where *q* is the equivalent stress, *p* is the hydrostatic stress, β is the friction angle, and *d* is the cohesion of the material. β represents the dependence on the stress triaxiality. Thus, the yield criterion becomes equivalent to the von Mises criterion, which does not account for stress triaxiality, when β=0. The mechanical properties depending on stress triaxiality can be determined through mechanical tests of adhesives under various loading conditions, such as tensile, shear, and compression tests. Figure 2 presents a comparison between experimental results and FE simulations using the linear Drucker–Prager yield criterion. The yield behavior influenced by stress triaxiality is accurately captured by the applied yield criterion.

**Figure 11:**
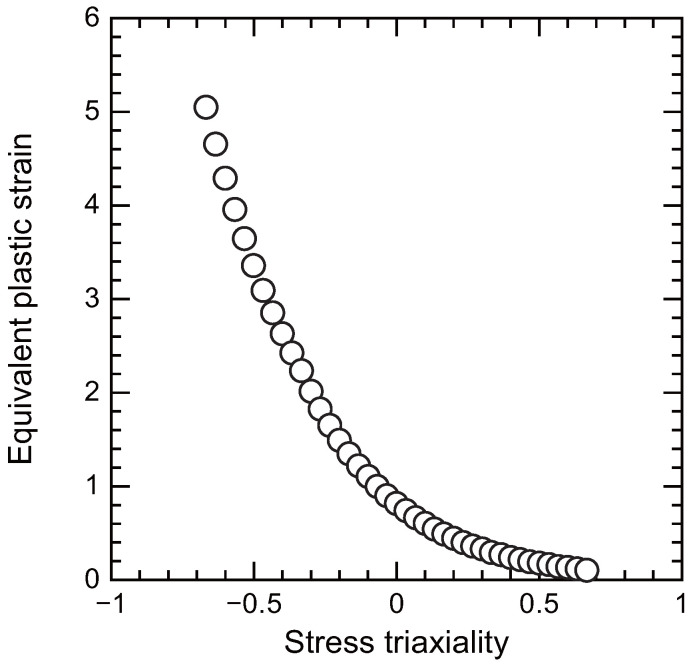
Relationship between equivalent plastic strain at failure and stress triaxiality (adopted from [114]).

Similar to yield criteria, stress triaxiality-dependent failure criteria have also been proposed. The Johnson–Cook failure model was initially developed for ductile metals and has since been applied to ductile adhesives. This model accounts for the influences of strain rate and temperature. However, when these effects are ignored, the Johnson–Cook failure criterion, which depends only on stress triaxiality, is expressed as [113]:

(2) $$\varepsilon_{f}=\left[D_{1}+D_{2}\exp\left(-D_{3}\frac{p}{q}\right)\right],$$

where $\varepsilon_{f}$ represents the equivalent plastic strain at the onset of damage, and $D_{1}$, $D_{2}$, and $D_{3}$ are material constants. Figure 11 illustrates an example of a stress triaxiality-dependent failure criterion [114]. The combination of the Drucker–Prager yield criterion and the Johnson–Cook failure criterion is commonly referred to as the toughened adhesive polymer (TAPO) material model [115,116].

The CZM is also widely used to analyze the progressive damage of adhesively bonded joints [117,118,119,120]. While the CZM can simulate crack propagation with a simpler formulation, it requires the resultant behavior of joints as input material properties. Consequently, the effects of local factors at the meso- to microscale, such as stress triaxiality and bondline thickness, cannot be directly simulated using the CZM. In contrast, the aforementioned continuum approach, such as the TAPO material model, can account for these effects, although it has a more complex formulation than that of the CZM.

**Figure 12:**
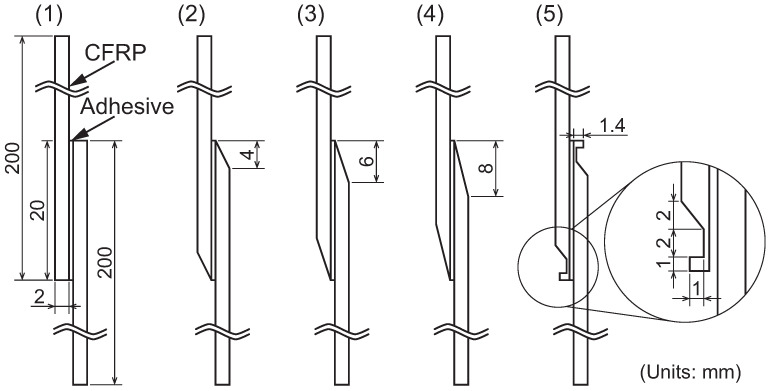
SLJs with different geometries (adopted from [114]).

Some previous studies utilizing stress triaxiality-dependent yield and damage models are reviewed herein. Because adherends constrain the adhesive layers, their shapes significantly influence stress triaxiality. In particular, stress concentration at the edges of joints is unavoidable. To mitigate stress concentration, tapered joints are commonly employed [121]. The effects of adherend shapes and bondline thickness in SLJs are simulated using FEM in Ref. [114]. The simulated SLJs are illustrated in Figure 12, where the simulations incorporate the stress triaxiality-dependent failure criterion shown in Figure 11.

**Figure 13:**
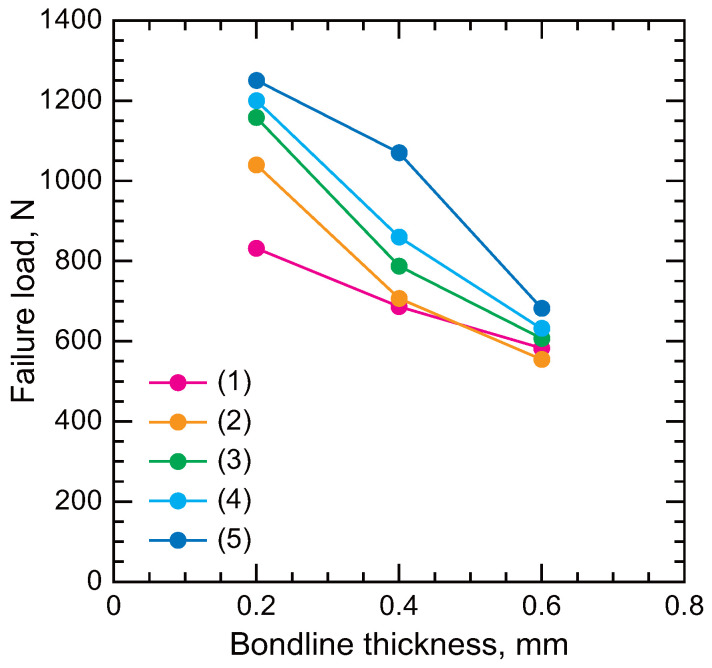
Failure load of SLJs obtained by FEM (adopted from [114]).

Figure 13 illustrates the variation in failure loads as a function of adherend shapes and bondline thickness in the SLJs [114]. The specimen numbers correspond to those shown in Figure 12. Failure loads for tapered joints were generally higher than those of SLJ (1), which lacks a taper, except for SLJ (2) with a 0.6 mm thick bondline. Furthermore, SLJ (5), featuring “humps” at the ends of the adherends, exhibited superior strength compared to simple tapered joints.

**Figure 14:**
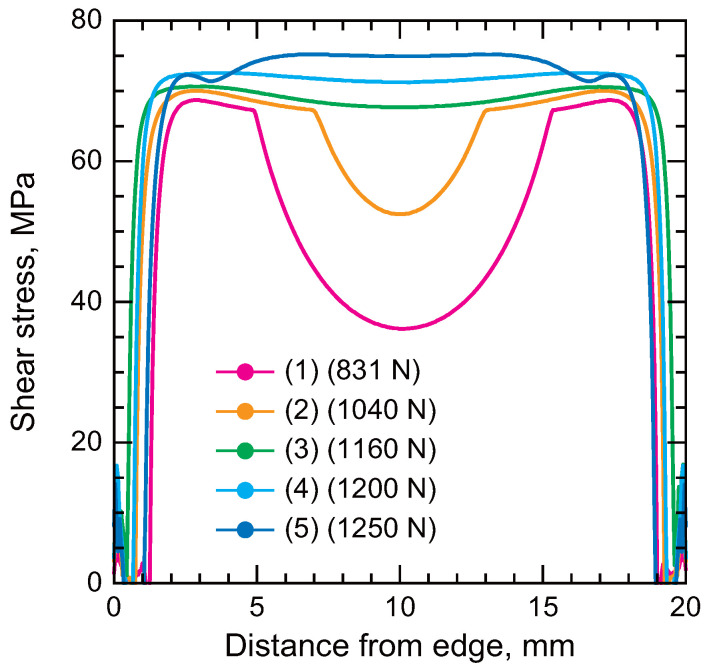
Shear stress distribution of SLJs immediately before final failure (adopted from [114]).

Figure 14 illustrates the shear stress distribution in the adhesive layers of SLJs with a 2 mm thick bondline immediately before final failure [114]. In SLJ (1) without taper, the shear stress is unevenly distributed, leading to premature failure. In contrast, SLJ (5) exhibits higher shear stress in the middle region compared to the edges. Since the stress triaxiality in the middle region is lower than that at the edges, SLJ (5) can withstand a higher tensile load compared to the other SLJs. As demonstrated by the simulation results, stress triaxiality-dependent behavior is a critical factor in joint design.

**Figure 15:**
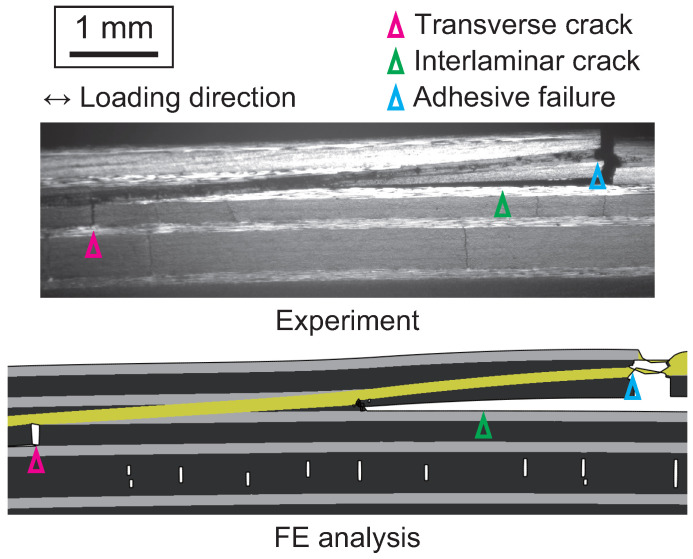
Comparison of failure morphology observed during experiment and obtained by FEM (adopted from [28]).

In adhesively bonded composite joints, the interaction between adherend and adhesive damage must be considered. Figure 15 compares the failure morphology of adhesively bonded composite scarf joints between experimental results and FE simulation [28]. In this FEM analysis, failure in the transverse layers of CFRP laminates is simulated using the continuum damage model, while failure at the ply interfaces is simulated using the CZM. By employing these models, the FEM accurately reproduces the failure process. FE analysis without incorporating composite failure models overestimated the failure stress, whereas the inclusion of these models provided a precise simulation of the failure stress [28].

Fatigue damage modeling is also critical for practical applications, as adhesively bonded structures often fail under cyclic loading. Similar to modeling under static loading, CZM-based fatigue damage models have been developed and widely utilized [122,123]. However, microscopic phenomena occurring within adhesive layers (e.g., crack kinking) cannot be accurately simulated using the CZM-based approach. To address this limitation, several continuum fatigue damage models have been proposed. In these models, determining the parameters that control material degradation during cyclic loading is essential. Plastic strain range [124], thermodynamic parameters [125,126,127], and entropy [128] are commonly used as control parameters for fatigue damage evolution.

Because polymers degrade under extreme environments [129,130,131], several models have been proposed to account for the effects of aging and hygrothermal conditions on the behavior of adhesively bonded joints. Aging and hygrothermal conditions not only cause degradation but can also lead to improvements in specific properties. For instance, an increase in failure strain has been reported after hygrothermal environmental conditioning, even though ultimate stress and Young’s modulus typically decrease [131,132]. Therefore, parameters for numerical simulation related to deformation and damage progression (e.g., yield stress, strain at failure, fracture toughness) are often calibrated to accurately capture the effects of aging and hygrothermal conditions [133,134,135].

## 5. Concluding Remarks and Future Perspectives

This article reviews the microscopic aspects of damage and failure in adhesively bonded composite joints. Because adhesives exhibit unique deformation and damage behaviors, the peculiar characteristics of polymeric materials are discussed in Section 2. Stress triaxiality significantly influences both yield and damage behaviors.

Section 3 reviews the experimental characterization of damage processes in adhesively bonded composite joints. In such joints, the microscopic damage process differs significantly from that in bulk materials due to the constraints imposed by the adherends. The effects of these constraints and various loading types are discussed based on the mechanics of polymers.

Section 4 introduces modeling and numerical simulations of adhesively bonded composite joints. Stress triaxiality-dependent yield and damage models are explained, demonstrating how high-fidelity progressive damage analyses can be performed using these models. The importance of the interaction between adhesive and adherend damage is also emphasized.

Thanks to recent advancements in measurement techniques, the microscopic damage processes in adhesively bonded composite joints have been increasingly revealed. However, direct optical measurements are limited to evaluating the surface of joints. High-resolution, non-destructive methods, such as SR X-CT, have seen growing application in recent years. Nevertheless, few studies have reported changes in strain fields associated with damage progression inside adhesively bonded joints. In particular, the effects of stress triaxiality in highly constrained regions (e.g., the center of joints along the width direction) have not been quantitatively evaluated to date. Therefore, a detailed microscopic damage evaluation inside joints, combining SR X-CT and DVC, is anticipated in the near future.

In addition, there have been only a few studies on the experimental characterization of microscopic damage progression during fatigue damage propagation. Although fatigue damage progression is primarily evaluated using indirect methods, direct in situ damage characterization under fatigue loading offers valuable insights into damage mechanisms. Given that high-magnification stationary observation is challenging even under static loading, the development of specialized testing equipment, such as trigger systems synchronized with a loading frame, is necessary. Microscopic direct observation can provide critical information, such as the effects of stress triaxiality, the development of the fracture process zone, and crack kinking. Since evaluating fatigue damage progression is crucial for practical applications, understanding damage progression at the microscale is essential for advancing our knowledge of damage mechanisms.

Considering the computational cost of sophisticated yield and damage models, simulating large-scale adhesively bonded structures poses significant challenges in terms of computational time. The damage process introduced in this review is an underlying phenomenon within the fracture process zone. Although the damage process can be simulated in coupon specimens, large-scale FE models are necessary to accurately replicate the failure behavior of large components, particularly due to the occurrence of damage in thin adhesive layers. To address this, transforming the intrinsic damage process into simplified models (e.g., CZM) is essential to effectively represent the underlying damage mechanisms while reducing computational complexity. Moreover, the interaction between adhesive damage and adherend damage in composite structures adds further complexity to simulation strategies. Overcoming these challenges is crucial for accurately modeling the damage process in large composite structures.

Another significant challenge lies in the lack of fatigue damage models at the microscale. As reviewed in this article, several promising models have been developed to simulate fatigue damage progression. However, most previous studies primarily focus on fatigue life prediction rather than the detailed progression of fatigue damage within the fracture process zone. Fatigue damage progression is influenced by various factors, including stress triaxiality, strain rate, and strain range. Constructing microscopic fatigue damage models that account for these complex effects in polymers is particularly challenging. Given the importance of evaluating fatigue damage progression for practical applications, understanding fatigue damage mechanisms at the microscale is essential for developing accurate fatigue damage models. In particular, the extent of microscopic damage progression (e.g., microscopic adhesive degradation) within the fracture process zone should be simulated and validated against experimental observations. Additionally, coupling analyses of adhesive damage and composite damage under cyclic loading remain an unexplored and challenging research area. The integration of fatigue damage models for both adhesives and composites could provide valuable insights into the safe and efficient design of composite structures.
